# The incidence of all stroke and stroke subtype in the United Kingdom, 1985 to 2008: a systematic review

**DOI:** 10.1186/1471-2458-10-539

**Published:** 2010-09-08

**Authors:** Prachi Bhatnagar, Peter Scarborough, Nigel C Smeeton, Steven Allender

**Affiliations:** 1 Department of Public Health, University of Oxford, Old Road Campus, Headington, Oxford, OX3 7LF, UK; 2 King's College London, Division of Health and Social Care Research, 7th Floor, Capital House, 42 Weston Street, London, SE1 3QD, UK

## Abstract

**Background:**

There is considerable geographic variation in stroke mortality around the United Kingdom (UK). Whether this is due to geographical differences in incidence or case-fatality is unclear. We conducted a systematic review of high-quality studies documenting the incidence of any stroke and stroke subtypes, between 1985 and 2008 in the UK. We aimed to study geographic and temporal trends in relation to equivalent mortality trends.

**Methods:**

MEDLINE and EMBASE were searched, reference lists inspected and authors of included papers were contacted. All rates were standardised to the European Standard Population for those over 45, and between 45 and 74 years. Stroke mortality rates for the included areas were then calculated to produce rate ratios of stroke mortality to incidence for each location.

**Results:**

Five papers were included in this review. Geographic variation was narrow but incidence appeared to largely mirror mortality rates for all stroke. For men over 45, incidence (and confidence intervals) per 100,000 ranged from 124 (109-141) in South London, to 185 (164-208) in Scotland. For men, premature (45-74 years) stroke incidence per 100,000 ranged from 79 (67-94) in the North West, to 112 (95-132) in Scotland. Stroke subtype data was more geographically restricted, but did suggest there is no sizeable variation in incidence by subtype around the country. Only one paper, based in South London, had data on temporal trends. This showed that there has been a decline in stroke incidence since the mid 1990 s. This could not be compared to any other locations in this review.

**Conclusions:**

Geographic variations in stroke incidence appear to mirror variations in mortality rates. This suggests policies to reduce inequalities in stroke mortality should be directed at risk factor profiles rather than treatment after a first incident event. More high quality stroke incidence data from around the UK are needed before this can be confirmed.

## Background

Stroke is one of the largest health burdens in the UK. In 2007, there were around 53,000 deaths from stroke and more than 175,000 consultant visits in National Health Service (NHS) hospitals [1]. Stroke also imposes an economic burden to the UK, costing about £7 billion a year, of which £2.8 billion are direct costs to the NHS [1]. Although stroke mortality rates in the UK have been falling steadily since the late 1960s, the burden of stroke may increase in the future as a consequence of the UK's ageing population [2].

This burden is not distributed equitably around the UK, with higher mortality rates in the North of England, Wales and Scotland compared to the South of England. The difference in mortality rates can be considerable, with premature mortality rates (deaths before age 75) in Scotland over 80% higher than in the South East of England in 2007 [3]. It is unclear how much of these differences in mortality rates are due to differences in stroke incidence (and hence the behavioural risk factor profile of populations), case fatality rates (and hence standards of treatment) or both. It is also unclear whether these geographic differences in the burden of stroke are due to different stroke subtypes, which differ in their established risk factors - the major risk factors for ischaemic stroke are smoking and hypercholesterolaemia, contributing to atheroma, whereas the major risk factor for haemorrhagic stroke is hypertension. Variations in the risk factor profiles of populations could therefore result in geographic variations in incidence of stroke by subtype. Routinely collected death certificate data are not complete enough to allow for an accurate description of geographic inequalities of stroke by subtype, as stroke deaths with the subtype not confirmed by autopsy or brain scan are recorded as unspecified stroke.

This paper reports on a systematic review of the published literature on the incidence of stroke in the UK between 1985 and 2008. The systematic review included studies of the incidence of all stroke, and also studies of the incidence of subtypes of stroke. The research questions informing this systematic review were:

1. How does the incidence of stroke vary geographically in the UK and how does it relate to geographic variations in stroke mortality?

2. Is the geographic pattern in stroke incidence mirrored by variation in the major subtypes of stroke - subarachnoid haemorrhage, haemorrhagic stroke and ischaemic stroke?

3. How has the incidence of stroke varied over time in the different areas of the UK?

## Methods

### Search Strategy

To identify papers both MEDLINE and EMBASE were searched, including all studies published between 1985 and 2008. Medical Subject Heading (MeSH) terms for "incidence", "stroke", "ischemic attack", "cerebrovascular disorders", "subarachnoid hemorrhage", "England", "United Kingdom", "Scotland", "Wales", and "Northern Ireland" were used. The search strategy is available from the authors on request. Duplicate papers were removed from the combined results of EMBASE and MEDLINE, using Reference Manager and a manual search. The reference lists of identified papers were searched for relevant studies, and the authors of studies that met the eligibility criteria (see below) were contacted and asked to identify any studies that may have been missed.

### Inclusion criteria

The inclusion criteria used for this review were based on those developed by Sudlow and Warlow [4], which were designed to identify high quality comparable incidence studies. These criteria are displayed below:

#### For studies of all stroke

• Stroke defined in the paper using either ICD-8 or ICD-9 430-438; ICD-10 I60-69 for cerebrovascular disease.

• Only studies of first cases of stroke included.

• Only prospective or retrospective study designs included.

• Study population and setting must be clearly defined, and within the United Kingdom.

• Study population must be the general population for the setting under study, rather than specific subset such as 'residential care patients'.

• Reasonable attempt must be made to identify all events in the study population (i.e. at least six monthly searching of databases of general practitioners in the study area for all patients coded with a cerebrovascular diagnosis.)

• Stroke events must be followed up for re-occurrence within individuals within the study period, using linked and overlapping data sources (e.g. GP and hospital records).

• Age and gender specific estimates must be recorded.

• Data collection period must cover at least one year.

#### Inclusion criteria for stroke subtypes

In addition to the criteria above, the inclusion criteria for papers by stroke sub type were as follows:

• Stroke subtypes defined by ICD codes, using the following definitions: Subarachnoid haemorrhage (ICD-8 or ICD-9 430; ICD-10 I60); Haemorrhagic stroke (ICD-8 431; ICD-9 431-432; ICD-10 I61-62); Ischaemic stroke (ICD-8 432-434; ICD-9 433-434; ICD-10 I63).

• At least 80% of events must have been categorised to stroke subtype by use of autopsy or brain scanning [5].

Only papers reporting original data (i.e. not reviews) were included in this review.

Where two papers meeting the inclusion criteria reported the same data over the same data collection period only the paper reporting the more detailed breakdown of the data collection period was retained. For example a paper reporting the time periods 1995-2004 in 2 year time bands [6] was favoured over papers from the same study population reporting the time periods 1995-96 [7] and 1995-2004 [8] combined.

Incidence derived from the included studies were compared against stroke mortality rates from the same time period and locality, derived using mortality data provided by the Office for National Statistics (ONS) and General Register Office for Scotland (GROS) [9]. Mortality rates were calculated for those aged 45 and over and 45 to 74 for accurate comparison to the reported incidence rates.

### Analysis

Since the aim of the paper required a comparison of results, no meta-analysis was attempted. Standardised results were prepared for comparison between studies, using either the data reported in the original studies or raw data attained by contacting the corresponding author of the included studies. Annual incidence per 100,000 population were derived for men and women in the age groups 45+ and 45-74 (defined here as 'premature incidence'). Results were directly standardised to the European Standard Population [10]. 95% confidence intervals for the incidence were calculated but should be treated with caution. Confidence intervals are designed to reflect sampling variation and rates reported here are based on all incident strokes within a population.

## Results

**Figure 1:**
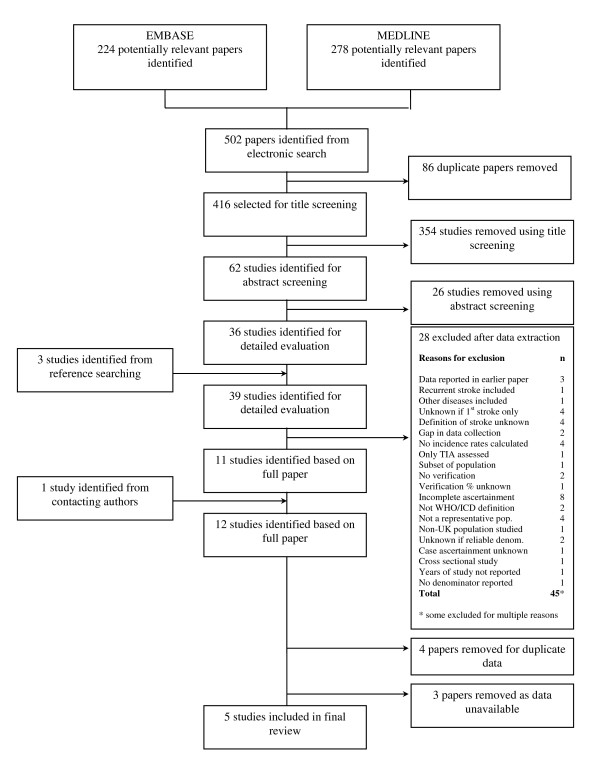
**Flow chart of the reviewing process**.

From the 502 papers identified in the initial search, five [6,11-14] were retained as meeting all of the inclusion criteria (figure 1). After examination of titles and abstracts, the most common reason for exclusion was incomplete ascertainment methods (eight studies excluded for this reason). Other common reasons for exclusion included not having a study population representative of the area, no definition of stroke reported and not reporting whether recurrent stroke was included. Nine papers were excluded having failed one of the inclusion criteria, while eighteen were excluded for multiple reasons.

**Figure 2:**
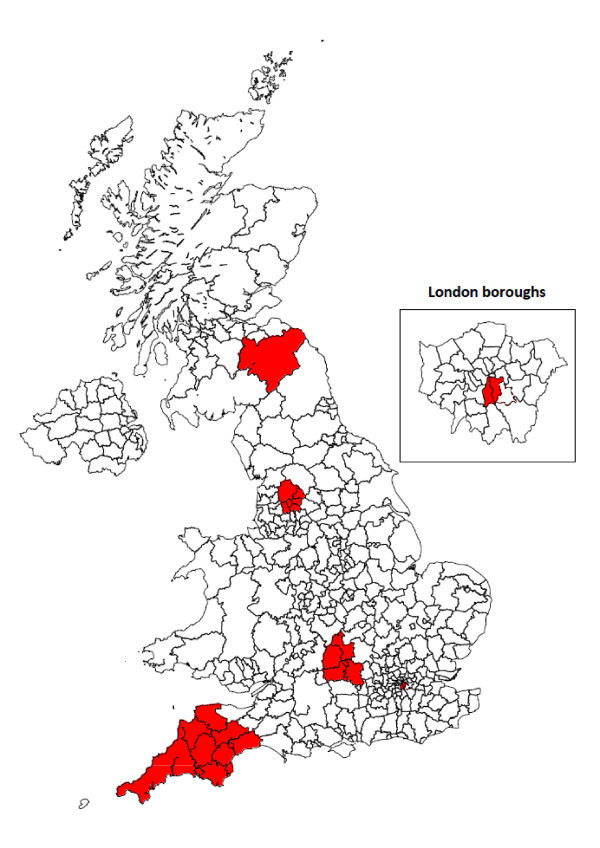
**Map showing areas of the UK covered by the included studies**.

Two of the five included studies were set in South East England (Oxfordshire, South London) and the remaining studies were set in Devon/Cornwall, East Lancashire and the Scottish Borders (table 1 and figure 2). The earliest study provided data for 1992 and the most recent was set in 2005. The South London Stroke Register (SLSR) provided five estimates of stroke incidence for two-year time intervals between 1995/96 and 2003/04.

**Table 1:** Details of included studies

Setting	Population source	Study years	Number of strokes	Population	Study Design	ICD code	Reference
East Lancashire	93 GP practices	1994-1995	642	405,272	Prospective; GP contacts, Hospital registers, death certificates, rehabilitation Services	ICD 9 430-438	Du et al 1997

Oxfordshire	63 Family Physician Registries	2002-2005	439	91,106	Prospective;, GP visits, monthly practice listings, monthly referral listings, death certificates; imaging or autopsy	ICD-10 I60-69	Rothwell 2005

South London	Lewisham, Southwark & Lambeth	1995-2004	2,874	271,817	Prospective; multiple overlapping sources of information, death certificates; CT scan/MRI, necropsy, cerebrospinal fluid analysis	ICD-9 430-434 & 436 ICD-10 I60-69	Heuschmann et al 2008

Scottish Borders Region	39 GP practices	1998-2000	596	106,352	Prospective; Multiple notification sources - GPs, nursing staff, neurovascular clinic staff, neighbouring hospitals, social services, "Chest, Heart and Stroke, Scotland"; hot pursuit by research nurses; death certificates' CT scans	ICD 9 430-438 ICD 10 160-169	Syme et al 2005

Devon & Cornwall	Referrals to Neurosurgery department	1992-1996	800	1,504,847	Retrospective; Hospital records, death statistics, cerebral angiography database, surgery database, GP records; CT scans, necropsy	ICD-10 I60, I61.5, I61.9, I62, I62.1, I62.9, I67.1, I69-I69.2, I72.0, I72.9 and corresponding ICD-9 codes before 1994.	Pobereskin 2001

### Geographical Trends in Stroke Incidence

#### All Strokes

**Figure 3:**
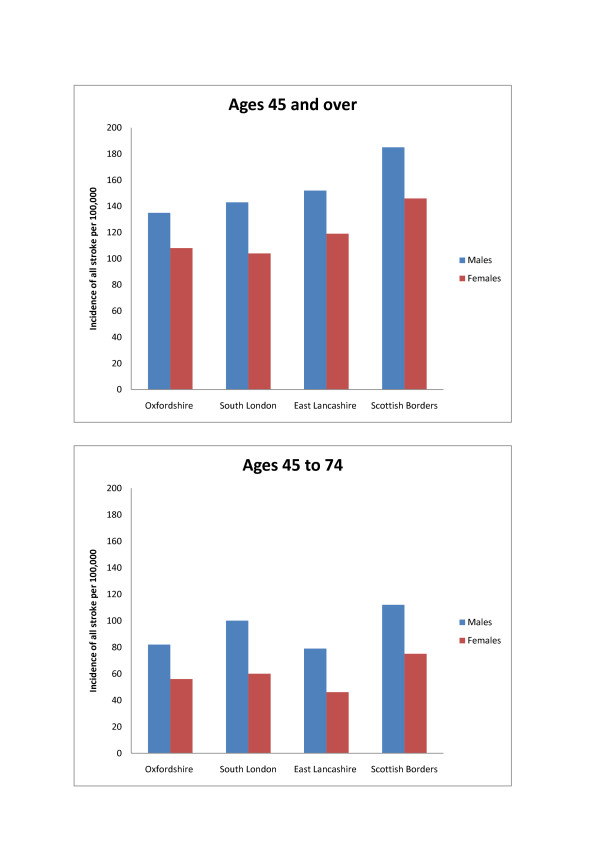
**Incidence of all strokes per 100,000, ages 45 and over and 45 to 74**.

Stroke incidence among men of all ages ranged from 124 per 100,000 in South London (2003/04) to 185 per 100,000 in the Scottish Borders region (1998/00) (table 2 and figure 3). Among women, incidence ranged from 88 to 146 per 100,000 in the same respective studies. Incidence in studies set in Oxfordshire and East Lancashire were similar, ranging from 135 to 152 per 100,000 among males.

**Table 2:** Incidence of all stroke and stroke subtypes, in men and women aged 45 and over

			Incidence/100,000 population (95% Confidence Interval)			
All stroke	Setting	Years	Males		Females	
Du et al 1997	East Lancashire	1994-1995	152.06	(134.43 - 172.00)	118.99	(107.36 - 131.88)
Syme et al 2005	Scottish Borders Region	1998-2000	184.78	(163.78 - 208.47)	145.76	(130.57 - 162.72)
Rothwell et al 2005	Oxfordshire	2002-2005	134.89	(117.63 - 154.67)	108.05	(94.65 - 123.36)
Heuschmann et al 2008	South London	1995-1996	150.20	(133.76 - 168.65)	115.20	(103.34 - 128.44)
		1997-1998	163.96	(146.71 - 183.23)	112.64	(96.56 - 120.42)
		1999-2000	134.94	(119.09 - 152.90)	93.93	(82.72 - 106.65)
		2001-2002	144.86	(128.28 - 163.59)	107.83	(99.84 - 127.08)
		2003-2004	124.16	(109.23 - 141.13)	87.52	(82.92 - 160.06)
**Ischaemic stroke**						

Rothwell et al 2005	Oxfordshire	2002-2005	120.93	(104.62-139.78)	91.63	(79.48 -105.63)
Heuschmann et al 2008	South London	1995-1996	111.58	(97.50 - 127.70)	79.93	(70.14 - 91.08)
		1997-1998	117.60	(103.20 - 134.02)	82.12	(72.12 - 93.50)
		1999-2000	99.24	(85.82 - 114.75)	73.44	(63.73 - 84.63)
		2001-2002	102.30	(88.50 - 118.25)	80.06	(69.79 - 91.83)
		2003-2004	99.24	(86.02 - 114.49)	70.27	(60.84 - 81.17)
**Intracerebral haemorrhage**						

Rothwell et al 2005	Oxfordshire	2002-2005	10.88	(6.85 - 17.27)	7.61	(4.66 - 12.42)
Heuschmann et al 2008	South London	1995-1996	19.20	(13.91 - 26.50)	15.01	(11.01 -20.47)
		1997-1998	28.67	(21.85 - 37.63)	12.49	(8.93 - 17.49)
		1999-2000	25.53	(17.45 - 31.73)	10.17	(6.87 - 15.05)
		2001-2002	24.37	(18.20 - 32.64)	13.70	(9.58 - 19.60)
		2003-2004	14.21	(9.75 - 20.73)	8.64	(5.69 - 13.13)
						
**Subarachnoid haemorrhage**						

Pobereskin et al 2001	Devon and Cornwall	1992-1996	5.76	(4.19 - 7.93)	8.85	(8.08 - 9.70)
Rothwell et al 2005	Oxfordshire	2002-2005	3.01	(1.13 - 8.02)	8.94	(5.19 - 15.40)
Heuschmann et al 2008	South London	1995-1996	5.39	(2.90 - 10.02)	7.62	(4.80 - 12.09)
		1997-1998	5.69	(3.06 - 10.57)	3.41	(1.62 - 7.15)
		1999-2000	5.71	(3.07 - 10.61)	5.83	(3.23 - 10.52)
		2001-2002	9.84	(6.12 - 15.82)	6.83	(3.88 - 12.03)
		2003-2004	3.94	(1.88 - 8.27)	5.62	(3.19 - 9.90)

#### Stroke subtypes

Only the studies from Oxfordshire [14] and South London [6] met the inclusion criteria for ischaemic and haemorrhagic stroke (tables 2 and 3). A further study from Devon and Cornwall met the inclusion criteria for subarachnoid haemorrhage [12]. The incidence of ischaemic stroke was much higher than for the other subtypes in both Oxfordshire and South London, accounting for between 80% and 90% of all incident strokes. Incidence of haemorrhagic stroke was much lower for males in Oxfordshire as compared to the equivalent years for SLSR study (2003/04). Subarachnoid haemorrhage incidence was similar and all very low in Oxfordshire, SLSR, and Devon and Cornwall (less than 10 per 100,000 for both men and women).

**Table 3:** Incidence of all stroke and stroke subtypes, men and women aged 45 to 74

			Incidence/100,000 population (95% Confidence Interval)			
All stroke	Setting	Years	Males		Females	
Du et al 1997	East Lancashire	1994-1995	79.19	(66.98 - 93.63)	46.25	(38.30 - 55.85)
Syme et al 2005	Scottish Borders Region	1998-2000	111.94	(94.96 - 131.95)	74.65	(61.48 - 90.64)
Rothwell et al 2005	Oxfordshire	2002-2005	81.71	(68.12 - 98.02)	56.15	(45.04 - 70.00)
Heuschmann et al 2008	South London	1995-1996	102.76	(89.17 - 118.42)	64.87	(54.80 - 76.79)
		1997-1998	108.31	(94.13 - 124.64)	58.70	(56.10 - 80.60)
		1999-2000	97.31	(83.65 - 113.19)	57.67	(47.75 - 69.64)
		2001-2002	104.51	(90.16 - 121.15)	67.24	(49.15 - 70.09)
		2003-2004	87.38	(74.54 - 102.44)	50.88	(53.06 - 79.32)
**Ischaemic stroke**						

Rothwell et al 2005	Oxfordshire	2002-2005	73.70	(60.87- 89.24)	44.94	(38.56 - 52.37)
Heuschmann et al 2008	South London	1995-1996	76.06	(64.49 - 89.71)	45.99	(37.69 - 56.12)
		1997-1998	78.80	(66.88 - 92.83)	43.91	(35.63 - 54.11)
		1999-2000	71.21	(59.68 - 84.98)	42.46	(34.11 - 52.87)
		2001-2002	71.86	(60.09 - 85.94)	45.56	(36.74 - 56.49)
		2003-2004	67.86	(56.66 - 81.28)	39.21	(31.17 - 49.32)
**Intracerebral haemorrhage**						

Rothwell et al 2005	Oxfordshire	2002-2005	4.93	(2.35 -10.34)	3.50	(1.46 - 8.41)
Heuschmann et al 2008	South London	1995-1996	13.63	(9.21 - 20.17)	9.44	(6.02 - 14.80)
		1997-1998	20.51	(14.79 - 28.43)	7.01	(4.15 - 11.84)
		1999-2000	18.37	(12.99 - 25.98)	6.55	(3.72 - 11.53)
		2001-2002	18.35	(12.97 - 25.94)	10.49	(6.69 - 16.45)
		2003-2004	11.20	(7.22 - 17.35)	5.61	(3.10 - 10.12)
**Subarachnoid haemorrhage**						

Pobereskin et al 2001	Devon and Cornwall	1992-1996	5.16	(4.48 - 5.94)	7.69	(6.90 - 8.56)
Rothwell et al 2005	Oxfordshire	2002-2005	3.01	(1.13 - 8.02)	7.84	(4.22 - 14.57)
Heuschmann et al 2008	South London	1995-1996	5.39	(2.90 -10.02)	6.03	(3.43 -10.62)
		1997-1998	5.28	(2.75 - 10.14)	3.14	(1.41 - 7.00)
		1999-2000	4.87	(2.43 - 9.73)	5.56	(2.99 - 10.33)
		2001-2002	8.98	(5.42 - 14.90)	6.83	(3.88 - 12.03)
		2003-2004	3.53	(1.59 - 7.87)	4.46	(2.23 - 8.92)

### Comparison with mortality rates

**Figure 4:**
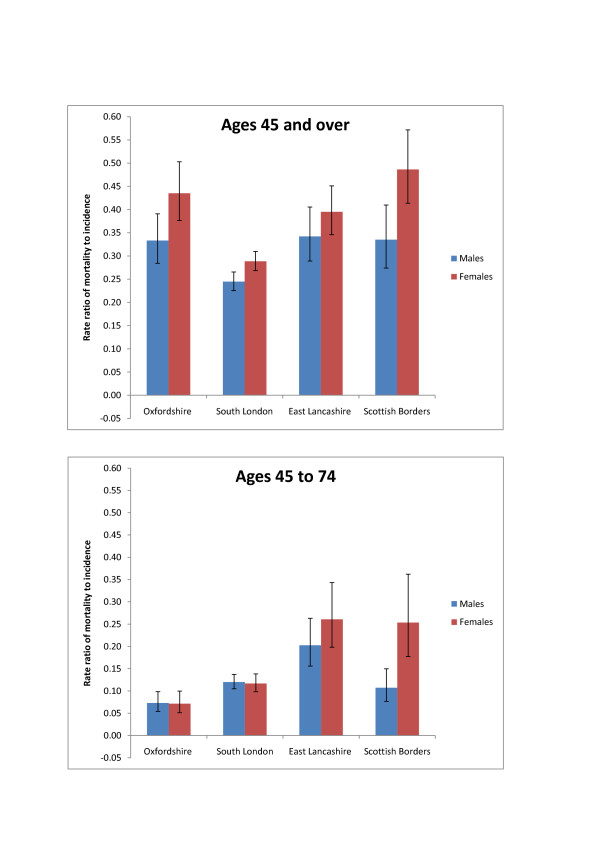
**Rate ratio of mortality to incidence for all strokes, with 95% confidence intervals**.

Rate ratios of mortality to incidence were used to compare the geographic variation in stroke incidence and mortality rates. For those aged 45 and over rate ratios were similar in East Lancashire, Oxfordshire and the Scottish Borders. However for the South London studies, the ratios for men and women were considerably lower than the other locations (0.24 for men and 0.29 for women in South London compared to 0.34 and 0.39 respectively in East Lancashire). For men aged 45 to 74 there was a higher mortality rate in East Lancashire compared to the other three locations. For women of the same age, the mortality rate in the two northern regions, East Lancashire and the Scottish Borders, was significantly higher than the mortality rate in the two southern regions (rate ratios of 0.26 and 0.25 in the northern regions compared to 0.07 and 0.12 in the south regions) (Table 4 and figure 4).

**Table 4:** Rate ratios of stroke mortality to incidence, ages 45 and over and 45 to 74

		Mortality rate		Incidence rate		Rate ratio of mortality to incidence			
Region	Year	Male	Female	Male	Female	Male	95% CI	Female	95% CI
**Ages 45 and over**									
Oxfordshire	2002-2005	45	47	135	108	0.33	(0.28 - 0.39)	0.44	(0.38 - 0.50)
South London	1995-2004	35	30	143	104	0.24	(0.23 - 0.27)	0.29	(0.27 - 0.31)
East Lancashire	1994-1995	52	47	152	119	0.34	(0.29 - 0.41)	0.39	(0.35 - 0.45)
Scottish Borders	1998-2000	62	71	185	146	0.34	(0.27 - 0.41)	0.49	(0.41 - 0.57)
									

**Ages 45 to 74**									
Oxfordshire	2002-2005	6	4	82	56	0.07	(0.05 - 0.10)	0.07	(0.05 - 0.10)
South London	1995-2004	12	7	100	60	0.12	(0.10 - 0.14)	0.12	(0.10 - 0.14)
East Lancashire	1994-1995	16	12	79	46	0.20	(0.16 - 0.26)	0.26	(0.20 - 0.34)
Scottish Borders	1998-2000	12	19	112	75	0.11	(0.08 - 0.15)	0.25	(0.18 - 0.36)

*Mortality rates are for the same years and location as the incidence rates.

†Incidence and mortality rates are age-standardised to the European Standard Population.

### Temporal trends in stroke incidence

From this review, there were only limited data on temporal trends in stroke incidence around the UK. The SLSR study was the only paper to have stroke incidence studies over more than one time period included in this review. While this did show a decline in incidence over the period 1995-2004, this cannot be compared to any other locations within the UK in this paper.

## Discussion

### Principal Findings

This systematic review included five studies of stroke incidence in a defined UK population between 1985 and 2008 that met established and accepted study quality criteria [4]. The small number of studies and the over-representation of the South of England only allowed for a limited investigation of the geographic variation in stroke incidence. The evidence identified in this review suggests that for those aged 45 and over, geographic patterns in stroke incidence largely mirror those of stroke mortality rates, with higher incidence and mortality in the north of the UK as compared to the south. For women under 75, there appears to be a higher case-fatality rate in the North of the UK compared to the South. The review also showed that not enough is known about geographic or temporal variations in the incidence of stroke (and stroke subtypes in particular) around the UK.

### Strengths and weaknesses

The inclusion criteria for the systematic review were based on a definition of an 'ideal' study of community incidence of stroke [5]. This ensured that only high quality, population based studies that had verified a large percentage of stroke cases were included in the review. One exception to the 'ideal' was the inclusion of a retrospective study. Retrospective studies of stroke incidence have the potential to underestimate incidence in a population because it is difficult to confirm whether all cases have been identified and verification of subtypes cannot be done particularly for the study, as they might otherwise have been in a prospective study. These issues are addressed in the other inclusion criteria for this review (for example, they must use overlapping methods of case ascertainment and have a high percentage of verified cases). Therefore retrospective studies included in this review are unlikely to have substantially underestimated the incidence. Differences in stroke incidence reported by the identified studies were most likely due to genuine differences in community incidence rather than differential biases introduced by study design.

The rigid inclusion criteria led to a limited number of studies being identified in the review. In particular, eight studies were excluded for not using multiple and/or overlapping case ascertainment methods. Between them these studies covered North East England, Scotland, East England, the South West of England and the whole of the UK. These studies would have widened the geographic range of the review, but their inclusion would have limited the comparability of the results.

It is likely that the systematic methodology used for this review produced a comprehensive set of stroke incidence studies in the UK between 1985 and 2008. Of the 36 studies identified for detailed evaluation, only four were not included in the original search of the MEDLINE and EMBASE databases. Three of these were identified from reference lists [15-17] and one was recommended by an expert in the field [6]. A subsequent search of both the MEDLINE and EMBASE databases established that the recommended paper had not been added to the databases at the time of the original search, but was added later.

The results presented in this review have been age-standardised to the European Standard Population to account for potential differences in the age structures of the populations being compared. This also allows a comparison to other studies standardising to this population. However, other population differences may also affect the comparability of the results. For example, the review included a study of a mostly White population in Devon and Cornwall, which contains both densely and sparsely populated areas, and an inner city population (South London) with a substantial ethnic mix [6,12]. It has been reported that Black people in South London have a higher incidence of stroke compared to Whites for all stroke and by subtype [18]. Of the major ethnic groups, Black Africans had the highest rates for intracerebral haemorrhage, and Black Caribbeans the highest rates for subarachnoid haemorrhage [8]. Additionally, the overall level of socio-economic deprivation of the areas included in the SLSR (Lewisham, Southwark and Lambeth) is greater than that found in much of Devon and Cornwall, and Oxfordshire. Given the strong associations between deprivation and many risk factors for both ischaemic and haemorrhagic stroke [19] it would be expected that the incidence of stroke would be higher in more deprived areas. Despite these differences in population characteristics, incidence of subarachnoid haemorrhage was similar in the SLSR and the study conducted in Devon and Cornwall.

Stroke incidence was reported for those aged 45 and above and those aged 45 to 74. The 45 to 74 age group included 49.5% of strokes overall, therefore geographic trends in premature stroke rates only account for half of all strokes that occur in the UK pattern. In general, premature stoke rates were supported by rates in strokes of all ages. The reporting of stroke incidence in the younger age group is common practice, and was included in the latest systematic review of worldwide stroke incidence [20]. Documenting the incidence of premature stroke in the UK is valuable for determining the extent to which premature stroke is a problem, as this may indicate a high prevalence of risk factors. Understanding the full pattern of stroke incidence will also aid prevention and treatment programs.

Three papers were excluded because the data reported in the studies did not allow for calculation of a standardised rate. The authors of these studies were contacted, but they were unable to provide the original data. Two papers reported incidence in Oxfordshire (Oxfordshire Community Stroke Project and Oxford Vascular Study) and one in South London (SLSR). The exclusion of these studies did limit the analysis of time trends in stroke incidence, but this is unlikely to have hindered the geographical analysis. The Oxfordshire Community Stroke Project had only one year of data that was eligible to be included in this review, from 1985 to 1986. The inclusion of this year would have extended the period for analysing time trends in stroke incidence by almost ten years, but the authors were unable to provide the raw data needed for our review. However, since these areas were represented by other studies included in the systematic review, their absence is unlikely to have affected the reporting of geographic variations in stroke incidence provided here.

The different time periods of the included studies should be considered when comparing incidence across the geographical regions. Stroke mortality rates have been declining since the late 1960 s [3] and it is likely that incidence has also declined, as a recent review on stroke worldwide [20] showed that there has been a 42% decrease in incidence over the past 4 decades, in high-income countries. It has also been reported that 50% of the recent decline in CHD mortality is due to reductions in the major risk factors, many of which are similar to those for stroke [21]. The included studies report incidence over more than a decade, from 1992 to 2005. Despite these differences in data collection periods, there did not appear to be a substantial difference in incidence between some of the different southern areas studied.

The adoption of the 10^th ^International Classification of by the UK in 2000 resulted in a 10% increase in reported stroke mortalities [22]. This review confirms that the increase in stroke mortalities is mirrored by an increase in the incidence of stroke (as would be expected).

### Comparison with previous findings

Five previous reviews [15-17,20,23] on the incidence of stroke have all reported on worldwide variations, and only the latest two reviews from 2009 [20,23] included more than one UK study. The three most recent of these reviews [17,20,23] used largely the same inclusion criteria as this paper [16]. Their international focus ignored geographic variations within the UK, which was the primary aim of this review. This is also the first systematic review to consider geographic patterns in incidence of stroke by subtype within the UK.

One of the latest systematic reviews on the incidence of stroke worldwide [20] supports the finding that changes in stroke mortality rates are most likely due to changes in incidence and risk factors, as stated in this review for those aged 45 and over. Explanations for differences in risk factor profiles do not come under the scope of this review, however associations between higher risk factor profiles and socioeconomic status [24] and some ethnicities [25] have been shown, with both of these factors varying greatly around the UK. The results found in this review therefore suggest that reducing inequalities in stroke mortality in the UK would best be achieved by tackling risk factors for stroke, namely hypertension, smoking and high alcohol consumption.

The results of this review suggest that stroke mortality rates in South London are lower than would be expected on the basis of incidence, for both men and women aged 45 and over. This may be due to the high number of ethnic groups included in the study population, as there is some evidence that Black people in South London have a higher survival rate after a stroke, as compared to White people [26]. The reasons for this are yet to be fully explained, but it has been postulated that differences in risk factor profiles, the 'healthy migrant' effect or type of stroke may be responsible, as there appears to be little difference in NHS stroke care between ethnic groups [27].

Given these potential differences in stroke mortality rates in different ethnic minorities, variation in subarachnoid haemorrhage incidence rates may have been expected between South London and Devon/Cornwall, due to the mainly White population in the latter and the high concentration of ethnic minorities in the former. There is some evidence that Black Africans have a stroke incidence rate similar to Whites [8] and in 2001, almost 90% of the SLSR population was White or Black African.

A higher case fatality rate in the North of the UK compared to the South was observed for those aged 45 to 74. This was particularly true for women. While higher case-fatality stroke rates for women compared to men have been reported worldwide [23], this does not offer explanations as to why there may be a difference between the northern and southern regions of the UK. General reasons for why case fatality may be higher in one place compared to another include a higher co-morbidity, more severe strokes, presenting at hospital later, or receiving differential treatment while in hospital. A paper examining sex differences in stroke burden in Scotland [28] also found higher case-fatality in women, but could offer no specific reason why this should be so in Scotland. The fact that the East Lancashire study has a higher case fatality in both sexes indicates that the explanation may lie in the date the study was carried out. The apparent North-South divide in case fatality in under 75 s (table 4) may be due to the time periods of data collection. The East Lancashire study was conducted in 1995. Since this time, in-hospital care for stroke has dramatically improved, with more than two-thirds of stroke patients now being admitted to a stroke unit [29].

## Conclusions

Geographic variations in stroke incidence in the UK appear to mirror variations in mortality rates with higher rates in the North of England and Scotland. More high quality stroke incidence data from around the UK are needed (particularly in Wales, Northern Ireland, Scotland and the central and northern areas of England) before this geographic variation can be confirmed.

## Competing interests

The authors declare that they have no competing interests.

## Authors' contributions

PB did the literature search, extracted the data and designed the tables and figures. PS and PB performed analyses. PB wrote the first draft and all authors subsequently helped write the article. All authors read and approved the final manuscript.

## Pre-publication history

The pre-publication history for this paper can be accessed here:

http://www.biomedcentral.com/1471-2458/10/539/prepub
